# Gender differences in stigma and care avoidance among people living with MASLD: An international survey

**DOI:** 10.1016/j.jhepr.2026.101987

**Published:** 2026-09-02

**Authors:** Patrizia Carrieri, Shira Zelber-Sagi, Saleh A. AlQahtani, Khalid Alswat, Yusuf Yilmaz, Caglayan Keklikkiran, Manuel Romero-Gómez, Jian-Gao Fan, Ming-Hua Zheng, Mohamed El-Kassas, Laurent Castera, Chun-Jen Liu, Vincent Wai-Sun Wong, Sombat Treeprasertsuk, Saeed Hamid, Hirokazu Takahashi, Takumi Kawaguchi, Ajay Duseja, Sven Francque, C. Wendy Spearman, Marlen I. Castellanos Fernández, Patrizia Burra, Stuart K. Roberts, Wah-Kheong Chan, Marco Arrese, Ashwani K. Singal, Stuart C. Gordon, Michael Fuchs, Naim Alkhouri, Rohit Loomba, Jane Ranagan, Janus P. Ong, Yuichiro Eguchi, Moises Diago, Ming-Lung Yu, Brian Lam, Lynn H. Gerber, Dana Ivankovsky-Wajcam, Linda Henry, Maria Stepanova, Zobair M. Younossi, Global NASH/MASH Council

**Affiliations:** 1 The Global NASH/MASH Council, Washington, DC, USA; 2 Aix Marseille Univ, Inserm, IRD, SESSTIM, Sciences Economiques & Sociales de La Santé & Traitement de L’Information Médicale, ISSPAM, Marseille, France; 3 School of Public Health, Faculty of Social Welfare and Health Sciences, University of Haifa, Haifa, Israel; 4 Liver, Digestive, and Lifestyle Health Research Section, and Organ Transplant Center of Excellence, King Faisal Specialist Hospital and Research Center, Riyadh, Saudi Arabia; 5 Liver Disease Research Center, College of Medicine, King Saud University, Riyadh, Saudi Arabia; 6 Department of Gastroenterology, School of Medicine, Recep Tayyip Erdoğan University, Rize, Turkey; 7 UCM Digestive Diseases and Ciberehd, Virgen Del Rocío University Hospital, Instituto de Biomedicina de Sevilla (HUVR/CSIC/US), Department of Medicine, University of Seville, Seville, Spain; 8 Department of Gastroenterology, Xinhua Hospital Affiliated to Shanghai Jiao Tong University School of Medicine, Shanghai, China; 9 MAFLD Research Center, Department of Hepatology, The First Affiliated Hospital of Wenzhou Medical University, Wenzhou, China; 10 Endemic Medicine Department, Faculty of Medicine, Capital University (Formerly Helwan University), Cairo, Egypt; 11 Department of Hepatology, Beaujon Hospital, AP-HP, Université Paris Cité, Inserm UMR1149, Clichy, France; 12 Hepatitis Research Center, Department of Internal Medicine and Graduate Institute of Clinical Medicine, National Taiwan University College of Medicine and Hospital, Taipei, Taiwan; 13 Department of Medicine and Therapeutics, Faculty of Medicine, The Chinese University of Hong Kong, Hong Kong, China; 14 Division of Gastroenterology, Department of Internal Medicine, Chulalongkorn University, Bangkok, Thailand; 15 Department of Medicine, Clinical Trials Unit, Aga Khan University, Karachi, Pakistan; 16 Liver Center, Saga University Hospital, Saga, Japan; 17 Division of Gastroenterology, Department of Medicine, Kurume University School of Medicine, Kurume, Japan; 18 Department of Hepatology, Post Graduate Institute of Medical Education and Research, Chandigarh, India; 19 Department of Gastroenterology and Hepatology, Antwerp University Hospital, Edegem, Belgium; 20 Division of Hepatology, Department of Medicine, Faculty of Health Sciences, University of Cape Town, Cape Town, South Africa; 21 Institute of Gastroenterology, University of Medical Sciences of Havana, Havana, Cuba; 22 Department of Gastroenterology, Padua University Hospital, Padua, Italy; 23 The Alfred Hospital and Monash University, Melbourne, VIC, Australia; 24 Gastroenterology and Hepatology Unit, Department of Medicine, University of Malaya, Kuala Lumpur, Malaysia; 25 Departamento de Gastroenterología, Facultad de Medicina, Pontificia Universidad Católica de Chile, Santiago, Chile; 26 Division of Gastroenterology and Hepatology, University of Louisville School of Medicine, Trager Transplant Center and Jewish Hospital, Louisville, KY, USA; 27 Department of Hepatology and Gastroenterology, Henry Ford Hospital System, Detroit, MI, USA; 28 Central Virginia VA Health Care System (CVHCS), Virginia Commonwealth University (VCU), Richmond, VA, USA; 29 Summit Clinical Research, San Antonio, TX, USA; 30 Division of Gastroenterology, University of California, San Diego, San Diego, CA, USA; 31 Chronic Liver Disease Foundation, Clark, NJ, USA; 32 College of Medicine, University of the Philippines, Manila, Philippines; 33 Loco Medical General Institute, Saga University Faculty of Medicine, Saga, Japan; 34 Hospital Quiron Salud, Valencia, Spain; 35 Hepatobiliary Division, Department of Internal Medicine, Kaohsiung Medical University Hospital, College of Medicine and Center of Metabolic Disorders and Obesity, Kaohsiung Medical University, Kaohsiung, Taiwan; 36 Center for Outcomes Research in Liver Diseases, Washington, DC, USA

**Keywords:** MASLD, Stigma, Discrimination, Liver-related comorbidities, Retention in care

## Abstract

**Background & Aims:**

Metabolic dysfunction-associated steatotic liver disease (MASLD) affects women and men differently, and both perceived and self-stigma may contribute to care avoidance. This study aimed to assess gender-specific patterns of stigma and their associations with care avoidance in MASLD.

**Methods:**

A cross-sectional, international survey was administered in 2023 by the Global NASH/MASH Council (GNC) among adults with a confirmed diagnosis of MASLD. The survey collected sociodemographic and clinical data and assessed perceived stigma and self-stigma using the validated Self-Stigma Scale–Short form, generating domain-specific and total scores. Severe self-stigma (simply termed self-stigma) was defined as a total score above the 75th percentile, stratified by gender. Care avoidance in primary or secondary care (gastroenterology, hepatology, and endocrinology) was reported.

**Results:**

Among 1,313 respondents with MASLD (51% men; 50% aged ≥55 years; 28% Europe, 26% USA/Canada, 30% Southeast/Southwest Asia, and 16% Middle East and North Africa), care avoidance was more frequent among men (23.6% *vs*. 18.9%, *p* = 0.04), occurring predominantly in primary care. Women reported higher self-stigma scores (all *p* <0.001) and more discrimination-related perceived stigma (27.8% *vs*. 19.1%). In adjusted analyses, both perceived stigma (adjusted odds ratio [OR] 1.84 [95% CI 1.07–3.18]) and self-stigma (adjusted OR 1.84 [95% CI 1.19–2.85]) were associated with care avoidance in men, whereas only perceived stigma was associated with care avoidance in women (adjusted OR 2.29 [95% CI 1.25–4.22]). In women, perceived stigma was linked to younger age, multimorbidity, and history of medical weight loss (*p* <0.05), whereas no predictors were identified in men. Lower socioeconomic status and advanced fibrosis predicted self-stigma in both genders.

**Conclusions:**

Stigma emerges as a major, gender-specific barrier to care in MASLD, with distinct mechanisms in men and women. Integrating gender-sensitive, stigma-informed strategies into MASLD care may improve access and continuity of care.

**Impact and implications:**

The presence of stigma arising from liver disease is associated with avoiding care. However, despite women reporting a higher degree of stigma than men, men more often avoided healthcare visits, especially when self-stigma was present. These differences in stigma and care avoidance by gender necessitate that healthcare providers who treat patients with MASLD develop gender-sensitive interventions to help ensure continued engagement with the healthcare system.

## Highlights

• Women with MASLD reported higher stigma across most dimensions, but men more often avoided healthcare visits.
• Perceived stigma roughly doubled the risk of care avoidance in both women and men with MASLD.
• Self-stigma independently predicted healthcare disengagement only among men with MASLD.
• Lower socioeconomic status and advanced fibrosis or cirrhosis were associated with higher self-stigma.
• Gender-sensitive, stigma-informed MASLD care may improve access and continuity of care.

## Graphical abstract

**Figure:**
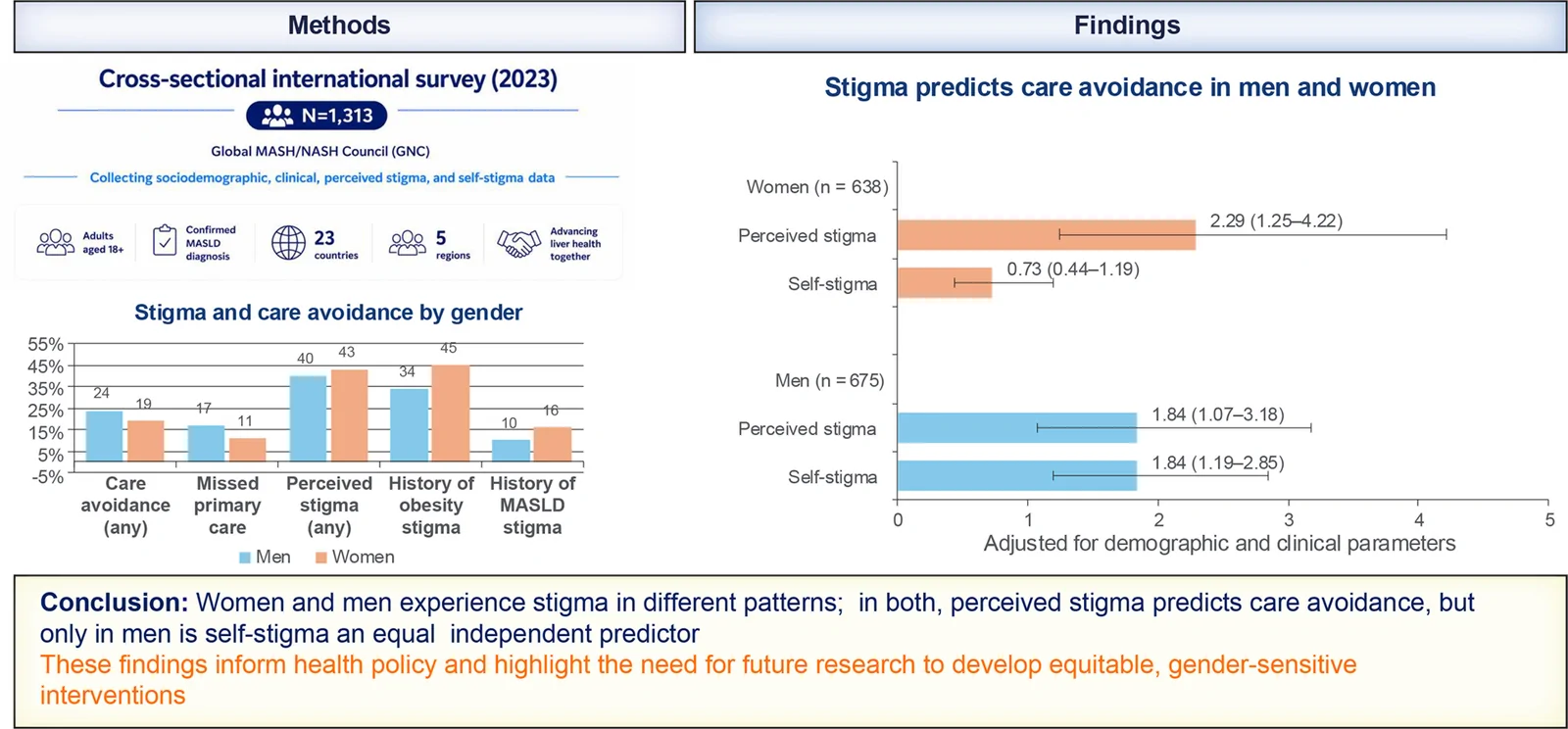

## Introduction

Metabolic dysfunction-associated steatotic liver disease (MASLD) affects men and women in distinct ways in terms of prognosis, physiological, mental, and social aspects. Gender-specific biological responses to obesity and metabolic dysfunction may modulate disease progression.^1^ Women have a higher risk of developing cirrhosis than men, and men have a higher risk of hepatic decompensation and more than double the risk of hepatocellular carcinoma among patients with MASLD.^2^^,^^3^ Furthermore, compared with age-matched men, postmenopausal women often exhibit shifts in fat distribution and metabolic risk profiles that may heighten their susceptibility to more advanced liver disease.^4^

Both genders may experience stigma and discrimination,^5^ which affects quality of life.

Stigma in chronic disease is commonly conceptualised as a multidimensional construct. In line with established models of stigma,^6^^,^^7^ it involves processes of labelling, stereotyping, and discrimination within a social context. We distinguish between perceived stigma and self-stigma. Perceived stigma refers to individuals’ perception of negative attitudes or discriminatory behaviours from others, including healthcare providers and society. Self-stigma refers to the internalisation of these attitudes, leading to feelings of shame, reduced self-esteem, and lower self-efficacy. Although distinct, these constructs are closely related and may influence healthcare behaviours differently, including care avoidance.^6^^,^^7^ In the Global NASH/MASH Council (GNC) survey completed by 1,976 patients with non-alcoholic fatty liver disease (NAFLD) across 23 countries, 26% reported having experienced (at least sometimes) stigma or discrimination because of being overweight/obese (ranging from 57% of enrollees from the USA to 7% of those from the Middle East and North Africa [MENA]), but only 8% experienced stigma because of their NAFLD (22% USA *vs*. 3% MENA). Furthermore, 26% of patients ever avoided a visit or missed a scheduled visit with a primary care physician because of NAFLD.^8^ A 2022 study showed that the majority (69%) of patients with NAFLD experience some form of stigmatisation in their daily lives.^9^ However, gender differences were not reported, and such data are lacking in the context of MASLD. Gender differences in different types of stigma and their relationship with care avoidance can be mostly learned from the field of obesity. Women tend to experience weight-related stigma^10^ more frequently and with greater emotional impact than men,^11^ especially among adolescents.^12^ This may be attributed to societal perceptions of body weight, body image, and chronic disease.^11^ Women with obesity often face more pronounced societal judgement and discrimination, which can contribute to greater psychological distress, reduced self-esteem, and poorer health-related quality of life.^13^ In contrast, although men with obesity also experience stigma, societal norms may be more accepting of excess weight in men, leading to differential psychosocial outcomes.^13^

Obesity-related stereotypes that are widespread across society lead to internalised weight stigma (also known as self-stigma), whereby individuals internalise negative societal attitudes and apply them to themselves. Internalised weight stigma can have a profound impact on psychological well-being and disease management. It may also contribute to disordered eating behaviours, further exacerbating metabolic health issues.^4^ Women tend to experience internalised weight stigma more than men.^10^ Although men may also experience self-stigma, this may be more linked to other factors, such as reduced perceived strength and control over their body.^14^ Internalised stigma can lead to feelings of guilt, shame, and self-blame, contribute to poor mental health, discourage individuals from seeking medical care, or reduce engagement in lifestyle interventions.^15^

Women with obesity are often more proactive in seeking medical care despite the stigma they experience.^16^ In a healthcare setting, women seem to experience more dismissive attitudes from their providers regarding their symptoms than men do, particularly when weight is involved.^17^ Conversely, among people with obesity, compared with women, men may delay seeking care until more severe complications arise, potentially leading to later-stage diagnoses.^18^ Poor social conditions may also attract more stigmatising and discriminatory attitudes, affect social cognition of individuals, and affect their understanding of the challenges of care, ultimately increasing both self-stigma and care avoidance.^19^^,^^20^

Although care avoidance affects a substantial proportion of individuals with MASLD,^9^ no study has assessed the relationship between stigma and care avoidance in individuals with MASLD and whether this relationship is modified by gender. In addition, among individuals with MASLD, it is unknown whether the pattern of determinants of stigma, particularly self-stigma, differs significantly by gender. This analysis aimed to shed light on the association of perceived stigma and self-stigma with care avoidance in men and women and to understand whether the pattern and predictors of stigma experience are gender specific. Understanding these nuanced differences is essential to strengthen the focus on patient-centred care, which can help tailor targeted interventions to reduce stigma, facilitate care, and improve care retention, thereby mitigating the overall burden of MASLD in both men and women.

## Materials and methods

### Study design and population

This cross-sectional study used data from an international survey developed and conducted through the GNC, which targeted individuals diagnosed with NAFLD/NASH who were recruited via GNC members.^5^ Recruitment flyers were developed for participating centres to use in their recruitment efforts. GNC members distributed the survey link to patients who expressed interest in the study. No incentives were used to recruit participants. Apart from a confirmed diagnosis of NAFLD or NASH and the ability and willingness to complete the survey, the study protocol did not define additional inclusion or exclusion criteria for patients. Following approval from their respective institutional review boards or equivalent regulatory bodies, council members shared the survey electronically with colleagues and/or patients in each participating centre. The survey link included an informed consent form; participation was anonymous, with no identifying information collected. The study was approved by the WIRB Copernicus Group (WCG); however, some participating institutions also required approval by their internal institutional review boards, which was sought and granted before the study commenced. All procedures adhered to the principles of the Declarations of Helsinki and Istanbul. The online survey platform allowed participants to skip individual questions, as responses were not mandatory. Only respondents who completed the survey and submitted it electronically (regardless of item-level missingness) were included in the analysis. Although the original survey used NAFLD/NASH terminology, all analyses in the present study were harmonised and interpreted according to the current MASLD/MASH nomenclature.

### Survey questionnaire

The survey comprised 68 items, including several multipart questions. The questionnaire gathered information on participants’ demographic and selected socioeconomic characteristics, liver fibrosis stage (if known), obesity status or history of obesity, and presence of comorbidities. In addition, it included scales for measuring self-stigma^21^ and perceived stigma^9^^,^^22^ and self-reported history of care avoidance (with a primary care physician, liver specialist, and/or endocrinologist).

The nine-item Self-Stigma Scale–Short (SSS–S) was adapted to MASLD (by replacing the name of the disease across the items). The SSS-S and the perceived stigma questionnaire were previously validated in their original versions and are widely used in populations with chronic diseases. The French version used in this study^21^ was translated into English using a standard forward translation process. No additional modifications were made beyond linguistic translation and adaptation to MASLD terminology**.** This allowed calculating a global score and three sub-scores (Affective, Cognitive, and Behavioural; each included three items) by adding up the responses quantified on a Likert scale (1 = ‘strongly disagree’, 2 = ‘disagree’, 3 = ‘neither agree nor disagree’, 4 = ‘agree’, and 5 = ‘strongly agree’). The domains of the SSS-S showed acceptable to good internal consistency (Cronbach’s alpha >0.66 for all three domains and >0.80 for the Cognitive and Behavioural domains).

The questionnaire used for the assessment of perceived stigma included 18 stigma-related questions that were categorised into four domains (Stereotypes, Discrimination, Shame, and Social Isolation), and answers to the questions were made on a 4-point Likert scale: ‘strongly disagree’, ‘disagree’, ‘agree’, and ‘strongly agree’. A participant was considered positive for perceived stigma in a particular domain if the answer to at least one question from the domain was ‘agree’ or ‘strongly agree’; a participant was considered positive for MASLD-related stigma if they were positive for perceived stigma in at least one domain.^9^

The survey was administered via a secure web-based link, ensuring full anonymity and the absence of any personally identifiable information. All completed survey data were stored in a centralised, secure database for analysis in aggregate.

### Statistical analysis

A descriptive analysis compared stigma and care avoidance experiences and sociodemographic and clinical characteristics between men and women with MASLD. Frequencies of categorical variables were expressed as counts (n) and percentages (%) and compared between groups using the Chi-square test. For continuous variables, comparisons were conducted using the non-parametric Mann–Whitney *U* test. All statistical tests were two-sided, and *p* <0.05 was considered statistically significant.

The three main study outcomes were as follows (any) care avoidance, perceived stigma (dichotomised), and self-stigma total score. Self-stigma total score was dichotomised to contrast people with values above the gender-specific 75th percentile (high self-stigma, simply termed self-stigma) with the others. A sensitivity analysis using the same gender-common 75th percentile as a cut-off for self-stigma was also performed. In the analysis of care avoidance predictors, perceived stigma and self-stigma were tested as the main potential explanatory variables. To model the influence of factors associated with care avoidance, perceived stigma, and self-stigma, mixed logistic regression models were used separately for women and men. Sociodemographic and clinical characteristics were considered as potential predictors of care avoidance, perceived stigma, and self-stigma. Predictors that returned a significant association with the respective outcome in a univariate model in at least one gender group were included in the respective multivariate models for both genders. Crude and adjusted odds ratios (ORs) were estimated from univariate and multivariate models, respectively. In the multivariate models, the geographical area was entered as a random-effect term to account for specific cultural effects. Interactions between each predictor and gender were also tested to determine whether the pattern of association differed between men and women.

Given the overall sample size and number of events per subgroup, the study is considered sufficiently powered to detect moderate associations (ORs in the range of approximately 1.5–2.0) between stigma measures and care avoidance, including in the smaller subgroup (women).

Data analyses were performed using SAS software, version 9.4 (SAS Institute Inc., Cary, NC, USA).

## Results

### Description of the study sample and comparison between genders in sociodemographic and clinical characteristics and outcomes

A total of 1,313 patients from 23 countries were included in this analysis. Participants were distributed as follows: 28% from Europe, 26% from the USA or Canada, 30% from Southeast or Southwest Asia, and 16% from the MENA region. Overall, 51% were men, 45% held at least a 4-year college degree, 39% resided in urban areas, 54% were employed, 13% reported liver cirrhosis, and 19% reported severe fibrosis (Table 1).

**Table 1:** Distribution of main sociodemographic and clinical variables, stigma, and care avoidance experience by gender (N = 1,313).

Demographic and clinical variables	All	Men	Women	*p* value
N	1,313	675	638	
Age (years)				
18–24	33 (2.5)	22 (3.3)	11 (1.7)	<0.001
25–34	99 (7.5)	72 (10.7)	27 (4.2)	
35–44	214 (16.3)	126 (18.7)	88 (13.8)	
45–54	311 (23.7)	177 (26.2)	134 (21.0)	
55–70	521 (39.7)	215 (31.9)	306 (48.0)	
70+	135 (10.3)	63 (9.3)	72 (11.3)	
BMI (kg/m^2^), mean ± SD	32.1 ± 9.4	32.1 ± 9.8	32.1 ± 9.0	0.13
Waist circumference (cm), mean ± SD	98.4 ± 15.2	98.9 ± 13.7	97.9 ± 16.8	0.28
Region				
Urban	509 (38.9)	270 (40.1)	239 (37.7)	0.60
Suburban	573 (43.8)	293 (43.5)	280 (44.2)	
Rural	226 (17.3)	111 (16.5)	115 (18.1)	
Geographic area				
Europe	373 (28.4)	213 (31.6)	160 (25.1)	<0.001
Middle East and North Africa	210 (16.0)	97 (14.4)	113 (17.7)	
Southeast Asia	305 (23.2)	160 (23.7)	145 (22.7)	
Southwest Asia	90 (6.9)	59 (8.7)	31 (4.9)	
USA/Canada	335 (25.5)	146 (21.6)	189 (29.6)	
Education				
None	70 (5.4)	18 (2.7)	52 (8.2)	<0.001
Primary school	223 (17.1)	93 (13.8)	130 (20.5)	
High school	422 (32.3)	229 (34.1)	193 (30.5)	
Bachelor	406 (31.1)	223 (33.2)	183 (28.9)	
Postgraduate	184 (14.1)	109 (16.2)	75 (11.8)	
Lives with a partner	962 (73.6)	526 (78.0)	436 (68.9)	<0.001
Employed	704 (53.9)	465 (69.1)	239 (37.8)	<0.001
Owner/renter of a home	899 (68.7)	450 (66.8)	449 (70.8)	0.11
Financial difficulties				
None	801 (61.4)	458 (68.2)	343 (54.2)	<0.001
Moderate	386 (29.6)	174 (25.9)	212 (33.5)	
High	118 (9.0)	40 (6.0)	78 (12.3)	
Has healthcare coverage	1,030 (78.7)	562 (83.5)	468 (73.7)	<0.001
Lost weight for medical reasons	547 (42.6)	250 (37.8)	297 (47.8)	<0.001
Other chronic conditions				
None	356 (27.4)	216 (32.4)	140 (22.2)	<0.001
One	386 (29.7)	211 (31.6)	175 (27.7)	
Two	274 (21.1)	135 (20.2)	139 (22.0)	
Three or more	283 (21.8)	105 (15.7)	178 (28.2)	
Diabetes or high glycaemia	550 (42.3)	267 (39.9)	283 (44.8)	0.08
Severe fibrosis^a^	116 (19.0)	73 (21.1)	43 (16.2)	0.12
Cirrhosis^a^	77 (12.6)	37(10.7)	40(15.0)	0.11
Current smokers	144 (11.0)	106 (15.8)	38 (6.0)	<0.001
Elevated alcohol use (AUDIT-C score ≥4 in men, 3 in women)	93 (7.5)	60 (9.4)	33 (5.5)	0.010
Care avoidance and stigma				
Ever missed a primary care physician visit	184 (14.2)	114 (17.2)	70 (11.0)	0.002
Ever missed an endocrinologist visit (if applicable)	130 (15.2)	64 (15.4)	66 (15.0)	0.86
Ever missed a gastroenterologist visit (if applicable)	137 (12.6)	75 (13.8)	62 (11.4)	0.24
Care avoidance (any, ever)	277 (21.3)	157 (23.6)	120 (18.9)	0.040
History of stigma because of				
MASLD	170 (13.1)	68 (10.2)	102 (16.1)	0.002
Overweight/obesity	512 (39.4)	226 (33.9)	286 (45.2)	<0.001
Type 2 diabetes	184 (14.2)	80 (12.0)	104 (16.5)	0.022
SSS-S				
Cognitive, mean ± SD	6.58 ± 3.19	6.29 ± 3.08	6.88 ± 3.28	<0.001
Affective, mean ± SD	6.62 ± 2.69	6.26 ± 2.52	7.00 ± 2.81	<0.001
Behavioural, mean ± SD	5.04 ± 2.00	4.82 ± 1.86	5.27 ± 2.12	<0.001
Total SSS-S, mean ± SD	18.2 ± 6.8	17.4 ± 6.5	19.1 ± 7.1	<0.001
High self-stigma^b^	338 (25.8)	166 (24.7)	172 (27.0)	NA
Perceived stigma				
Stereotypes	679 (51.9)	352 (52.3)	327 (51.4)	0.75
Discrimination	305 (23.3)	128 (19.1)	177 (27.8)	<0.001
Shame	756 (57.9)	372 (55.5)	384 (60.5)	0.07
Social isolation	523 (40.0)	252 (37.5)	271 (42.6)	0.06
Any perceived stigma	989 (75.5)	518 (77.0)	471 (73.9)	0.20

Data are presented as n (%), unless otherwise stated. Frequencies of categorical variables were compared between groups using the Chi-square test. For continuous variables, comparisons were conducted using the non-parametric Mann–Whitney *U* test.

MASLD, metabolic dysfunction-associated steatotic liver disease; NA, not applicable; SSS-S, Self-Stigma Scale–Short.

a Self-reported, available for 47% of respondents.

b High self-stigma was defined as the top quartile of the total SSS-S score, by gender (≥21 for men, ≥24 for women).

Women reported having experienced stigmatisation or discrimination related to MASLD, obesity, and diabetes significantly more commonly than men (all *p* <0.05). Self-stigma scores were also significantly higher in women across all domains (Cognitive, Affective, Behavioural, and total; *p* <0.001). However, no significant gender differences were found for any perceived stigma (*p* = 0.20) or for the specific subscales of Stereotypes, Shame, and Social Isolation (*p* >0.05), except for the Discrimination subscale (*p* <0.001). The prevalence of care avoidance was significantly higher among men than among women (23.6% *vs*. 18.9%, *p* = 0.040), primarily because of a higher rate of primary care avoidance among men.

### Predictors of care avoidance in men and women with MASLD

In univariable analyses (Table 2), among men, care avoidance was associated with high self-stigma, perceived stigma, younger age, employment, absence of chronic conditions, smoking, unknown fibrosis status (protective), and absence of type 2 diabetes. Among women, care avoidance was associated with perceived stigma, younger age, unknown fibrosis status (protective), and smoking.

**Table 2:** Predictors of care avoidance in men and women.

Predictors of care avoidance	OR (95% CI)^a^	*p*	AOR (95% CI)^a^	*p* value
**Men**				
High self-stigma^b^	1.86 (1.25–2.75)	0.002	1.84 (1.19–2.85)	0.006
Perceived stigma (any)	1.51 (0.96–2.38)	0.08	1.84 (1.07–3.18)	0.028
Age, per 10 years	0.83 (0.72–0.95)	0.007	0.97 (0.81–1.15)	0.72
Employed	1.65 (1.09–2.50)	0.018	1.35 (0.84–2.18)	0.22
No chronic conditions	1.72 (1.10–2.70)	0.018	1.37 (0.83–2.26)	0.22
One chronic condition	Reference		Reference	
Two or more chronic conditions	1.10 (0.69–1.74)	0.70	1.43 (0.85–2.40)	0.18
Type 2 diabetes	0.53 (0.36–0.78)	0.001	0.65 (0.41–1.05)	0.08
Advanced fibrosis or cirrhosis	0.79 (0.47–1.33)	0.37	0.83 (0.47–1.49)	0.54
No advanced fibrosis	Reference		Reference	
Unknown fibrosis status	0.65 (0.44–0.96)	0.030	0.70 (0.45–1.09)	0.12
Smoker	1.62 (1.02–2.56)	0.039	1.50 (0.91–2.46)	0.11

**Women**				
High self-stigma^b^	0.87 (0.55–1.38)	0.56	0.73 (0.44–1.19)	0.21
Perceived stigma (any)	1.85 (1.11–3.08)	0.018	2.29 (1.25–4.22)	0.008
Age, per 10 years	0.85 (0.71–1.01)	0.06	0.84 (0.68–1.05)	0.13
Employed	1.21 (0.81–1.82)	0.36	1.11 (0.69–1.78)	0.66
No chronic conditions	1.17 (0.66–2.07)	0.60	1.19 (0.63–2.23)	0.60
One chronic condition	Reference		Reference	
Two or more chronic conditions	1.18 (0.73–1.92)	0.49	1.30 (0.76–2.24)	0.33
Type 2 diabetes	1.08 (0.73–1.62)	0.70	1.25 (0.78–2.00)	0.36
Advanced fibrosis or cirrhosis	0.76 (0.41–1.43)	0.40	0.92 (0.47–1.83)	0.82
No advanced fibrosis			Reference	
Unknown fibrosis status	0.54 (0.35–0.84)	0.006	0.60 (0.37–0.97)	0.037
Smoker	2.08 (1.02–4.25)	0.045	2.33 (1.07–5.10)	0.033

AOR, adjusted odds ratio; OR, odds ratio.

a Crude ORs and AORs were derived from univariate and multivariate logistic regression models (with geographic region also included).

b AOR differed significantly between men and women (*p* <0.05 for interaction).

In multivariable mixed logistic regression models (Table 2), independent predictors of care avoidance differed by gender. Among men, only self-stigma (adjusted OR [AOR] 1.84 [95% CI 1.19–2.85]) and perceived stigma (AOR 1.84 [95% CI 1.07–3.18]) remained independently associated with care avoidance. Among women, perceived stigma (AOR 2.29 [95% CI 1.25–4.22]) and smoking (AOR 2.33 [95% CI 1.07–5.10]) independently predicted care avoidance, whereas unknown fibrosis status compared with no advanced fibrosis was associated with lower odds of care avoidance (AOR 0.60 [95% CI 0.37–0.97]). Despite significantly affecting more women than men, self-stigma was not associated with care avoidance in women, and this difference in the association with self-stigma in men and women was confirmed by a significant interaction between gender and self-stigma. No other predictors demonstrated statistically significant gender interactions in the adjusted analysis. The sensitivity analysis using the gender-common cut-off for the self-stigma total score (=23) confirmed the lack of association between self-stigma and care avoidance in women (AOR 0.73 [95% CI 0.45–1.18], *p* = 0.20).

### Predictors of self-stigma in men and women with MASLD

In univariable analyses (Table 3), among men**,** self-stigma was associated with rural residence, lack of home ownership, financial difficulties, and advanced fibrosis or cirrhosis. Among women, self-stigma was associated with financial difficulties, prior weight loss for medical reasons, presence of two or more chronic conditions, type 2 diabetes, and advanced fibrosis or cirrhosis.

**Table 3:** Predictors of self-stigma in men and women with MASLD.

Predictors of severe self-stigma	OR (95% CI)^a^	*p*	AOR (95% CI)^a^	*p* value
**Men**				
Region: rural (ref: suburban or urban)	1.59 (1.02–2.48)	0.040	1.68 (1.03–2.74)	0.037
Owner/renter of a home	0.64 (0.44–0.91)	0.015	0.58 (0.39–0.87)	0.008
No financial problem	0.61 (0.42–0.88)	0.008	0.74 (0.50–1.11)	0.14
Ever lost weight for medical reasons	1.42 (0.99–2.04)	0.06	1.25 (0.84–1.87)	0.27
No chronic conditions	0.75 (0.47–1.19)	0.22	0.75 (0.45–1.25)	0.27
One chronic condition	Reference		Reference	
Two or more chronic conditions	1.28 (0.84–1.94)	0.26	1.05 (0.66–1.67)	0.85
Type 2 diabetes	1.35 (0.95–1.92)	0.10	0.94 (0.61–1.46)	0.78
Advanced fibrosis or cirrhosis	3.02 (1.83–4.99)	<0.001	2.73 (1.61–4.65)	<0.001
No advanced fibrosis	Reference		Reference	
Unknown fibrosis status	1.35 (0.89–2.04)	0.16	1.25 (0.81–1.94)	0.32

**Women**				
Region: rural (ref: suburban or urban)	1.11 (0.71–1.74)	0.65	0.91 (0.55–1.49)	0.7
Owner/renter of a home	0.75 (0.51–1.09)	0.13	0.71 (0.47–1.08)	0.11
No financial problem	0.58 (0.41–0.83)	0.003	0.61 (0.41–0.91)	0.014
Ever lost weight for medical reasons	1.67 (1.17–2.38)	0.005	1.52 (1.03–2.24)	0.037
No chronic conditions	1.05 (0.61–1.82)	0.85	0.93 (0.52–1.67)	0.81
One chronic condition	Reference		Reference	
Two or more chronic conditions	1.94 (1.26–2.99)	0.003	1.47 (0.90–2.40)	0.13
Type 2 diabetes	1.47 (1.03–2.09)	0.038	1.02 (0.67–1.55)	0.93
Advanced fibrosis or cirrhosis	2.54 (1.47–4.40)	0.001	2.45 (1.36–4.41)	0.003
No advanced fibrosis	Reference		Reference	
Unknown fibrosis status	1.03 (0.68–1.55)	0.90	1.78 (1.1–2.88)	0.019

AOR, adjusted odds ratio; MASLD, metabolic dysfunction-associated steatotic liver disease; OR, odds ratio.

a Crude ORs and AORs were derived from univariate and multivariate logistic regression models (with geographic region also included).

In multivariable mixed logistic regression models (Table 3), independent predictors of self-stigma differed by gender. Among men, rural residence was independently associated with higher odds of self-stigma (AOR 1.68 [95% CI 1.03–2.74]), whereas home ownership was associated with lower odds (AOR 0.58 [95% CI 0.39–0.87]). Advanced fibrosis or cirrhosis remained strongly associated with self-stigma (AOR 2.73 [95% CI 1.61–4.65]). Among women, absence of financial problems was independently associated with lower odds of self-stigma (AOR 0.61 [95% CI 0.41–0.91]), whereas prior weight loss for medical reasons was associated with increased odds (AOR 1.52 [95% CI 1.03–2.24]). Advanced fibrosis or cirrhosis was independently associated with self-stigma (AOR 2.45 [95% CI 1.36–4.41]), as was unknown fibrosis status (AOR 1.78 [95% CI 1.10–2.88]) compared with no advanced fibrosis.

No significant effect modification by gender was detected for any predictor of high self-stigma in the multivariable models (all gender-by-predictor interaction *p* values ≥0.05).

### Predictors of perceived stigma in men and women with MASLD

In univariable analyses (Table 4), among men, perceived stigma was associated with older age, college education, home ownership, having two or more chronic conditions, and advanced fibrosis or cirrhosis. Among women, perceived stigma was associated with college education, employment, prior weight loss for medical reasons, and absence of chronic conditions (protective).

**Table 4:** Predictors of perceived stigma in men and women using univariable and multivariable logistic regression models.

Predictors of perceived stigma	OR (95% CI)^a^	*p*	AOR (95% CI)^a^	*p* value
**Men**				
Age, per 10 years	1.15 (1.00–1.33)	0.046	0.92 (0.76–1.11)	0.38
College degree	1.47 (1.03–2.12)	0.036	1.34 (0.87–2.06)	0.18
Employed	0.86 (0.58–1.28)	0.46	1.16 (0.71–1.89)	0.56
Owner/renter of a home	1.47 (1.01–2.12)	0.044	1.08 (0.69–1.70)	0.72
Ever lost weight for medical reasons^b^	1.43 (0.97–2.09)	0.07	1.13 (0.73–1.75)	0.58
No chronic conditions^b^	0.92 (0.60–1.42)	0.70	1.38 (0.83–2.31)	0.21
One chronic condition	Reference		Reference	
Two or more chronic conditions	1.64 (1.04–2.59)	0.035	1.21 (0.73–2.00)	0.47
Advanced fibrosis or cirrhosis	2.17 (1.20–3.91)	0.010	1.76 (0.91–3.42)	0.10
No advanced fibrosis	Reference		Reference	
Unknown fibrosis status	1.43 (0.97–2.10)	0.07	0.97 (0.61–1.54)	0.89

**Women**				
Age, per 10 years	0.90 (0.76–1.06)	0.19	0.77 (0.61–0.97)	0.026
College degree	1.75 (1.20–2.55)	0.004	1.28 (0.79–2.08)	0.32
Employed	1.75 (1.19–2.58)	0.004	1.33 (0.81–2.20)	0.26
Owner/renter of a home	0.85 (0.57–1.26)	0.41	0.84 (0.52–1.36)	0.48
Ever lost weight for medical reasons^b^	3.62 (2.43–5.41)	<0.001	2.67 (1.70–4.18)	<0.001
No chronic conditions^b^	0.41 (0.25–0.66)	<0.001	0.43 (0.24–0.78)	0.005
One chronic condition	Reference		Reference	
Two or more chronic conditions	1.13 (0.72–1.76)	0.60	0.78 (0.47–1.31)	0.35
Advanced fibrosis or cirrhosis	1.80 (0.94–3.43)	0.07	1.42 (0.65–3.13)	0.38
No advanced fibrosis	Reference		Reference	
Unknown fibrosis status	1.09 (0.74–1.62)	0.67	0.77 (0.46–1.29)	0.32

a Crude ORs and AORs were derived from univariate and multivariate logistic regression models (with geographic region also included).

b AOR differed significantly between men and women (*p* <0.05 for interaction).

In multivariable mixed logistic regression models (Table 4), independent predictors of perceived stigma differed by gender. Among men, no variables remained independently associated with perceived stigma after adjustment. Among women, younger age was independently associated with higher odds of perceived stigma (AOR 0.77 per 10-year increase [95% CI 0.61–0.97]). Prior weight loss for medical reasons remained strongly associated with perceived stigma (AOR 2.67 [95% CI 1.70–4.18]), whereas absence of chronic conditions was associated with lower odds (AOR 0.43 [95% CI 0.24–0.78]).

In the multivariable logistic regression analyses, two predictors showed significant effect modification by gender: having ever lost weight for medical reasons was strongly associated with higher odds of perceived stigma only among women, and having no chronic conditions was associated with lower odds of perceived stigma only among women; these associations were not observed in men.

## Discussion

This international survey highlights important gender differences in the experience and consequences of stigma among people living with MASLD. First, the prevalence of perceived stigma and self-stigma is significantly higher in women than in men. Although self-stigma was more prevalent among women, it appeared more deleterious in men, being independently associated with care avoidance, particularly avoidance of primary care consultations. Conversely, perceived stigma emerged as the main determinant of care avoidance in both men and women, with a comparable strength of association (approximately double the risk of care avoidance). These findings underscore the multidimensional nature of stigma and its potential to shape healthcare-seeking behaviours differently according to gender and social context. Taken together, these findings support a gender-differentiated stigma pathway in MASLD, whereby women experience a higher overall stigma burden, whereas men are more likely to translate internalised stigma into healthcare disengagement. This distinction highlights the importance of addressing not only the presence of stigma but also its gender-specific mechanisms when designing interventions to improve care retention.

The gender pattern observed in this study among patients with MASLD mirrors findings from other chronic diseases, where women tend to internalise stigma more deeply but remain more proactive in maintaining contact with healthcare services, possibly because of greater familiarity with health systems or higher engagement in preventive care.^16^^,^^23^^,^^24^ Men may experience self-stigma as a threat to their perceived strength, autonomy, or control over their body, which could explain their higher likelihood of care avoidance when stigma is internalised.^25^ In agreement with our study of the population with MASLD, men from the general population also tend to avoid or delay primary care to a greater extent than women, with lower GP consultation rates.^26^ Women often face multiple layers of social discrimination, encompassing weight, appearance, and disease-related stigma. Although this compounded stigma may elevate self-stigma levels, their resilience and coping strategies can mitigate its impact on healthcare-seeking behaviours and continuity of care.^27^

The finding that perceived stigma—rather than self-stigma—remains the main driver of care avoidance in both genders is particularly relevant for public health interventions. Perceived stigma reflects experiences of judgement, discrimination, and stereotyping by others, including healthcare professionals. This dimension may directly erode trust in the healthcare system, leading patients to delay or avoid care, as observed in other chronic diseases, such as obesity and mental health disorders.^28^ The association between perceived stigma and liver fibrosis or cirrhosis may also indicate that disease severity amplifies stigmatisation, either through visible symptoms or increased interactions with health services. Among women, perceived stigma was further linked to financial difficulties and comorbidities, suggesting that social vulnerability and multimorbidity reinforce each other in shaping stigmatising experiences.^29^

Notably, in men, no other sociodemographic or clinical factors were associated with care avoidance, emphasising that stigma—rather than disease characteristics or socioeconomic status *per se*—plays a central role in healthcare disengagement.

In women, two additional factors (liver fibrosis knowledge and smoking) were significantly associated with care avoidance. Lack of liver fibrosis knowledge was a protective factor against care avoidance, maybe because those with confirmed liver disease tend to avoid care—particularly primary care—because of fear of liver disease-related stigma.^9^ Unknown fibrosis status may also reflect lower disease awareness, which could attenuate anticipated stigma and reduce the perceived need to disengage from care.^30^ Conversely, this result is inconsistent with other research showing that awareness of disease severity may influence engagement with care.^31^ In women, smoking was independently associated with care avoidance. This association should be interpreted cautiously, as smoking may act as a proxy for broader vulnerability, including socioeconomic disadvantage,^32^ mental health comorbidity,^33^ and clustering of health-risk behaviours,^34^ all of which are linked to lower healthcare utilisation. Although smoking may also reflect anticipated judgement or stigma-related processes,^35^^,^^36^ these mechanisms cannot be directly assessed in this study. Further research is needed to disentangle these pathways.

Gender differences observed in this study likely reflect a combination of biological and sociocultural mechanisms. Although biological factors may contribute to differences in disease severity and clinical presentation of MASLD, our findings mainly highlight the role of sociocultural processes, particularly stigma and gendered patterns of healthcare engagement, as already demonstrated in other chronic diseases.^37^ These mechanisms may further intersect with clinical vulnerability and socioeconomic factors, suggesting a multifactorial and context-dependent nature of gender differences in MASLD.

Our results are consistent with previous studies. An online survey-based study among 857 adults with type 2 diabetes from the USA demonstrated that internalised weight and diabetes-related stigma strongly correlated with avoidance of healthcare, poor patient–provider communication, and reduced uptake of regular check-ups.^24^ Stigma in MASLD is associated with lower health-related quality of life,^5^ higher anxiety and depressive symptoms,^38^ and, in the case of weight stigma, unhealthy eating, alcohol use, and sleep disturbances.^39^ In ∼27,000 Spanish adults with obesity, internalised weight stigma was independently linked to higher MASLD risk, after adjusting for demographic, socioeconomic, and lifestyle factors.^40^

Notably, the care avoidance we measured was mainly reported in primary care. This finding is concerning in light of recent consensus and guideline recommendations calling for the greater involvement of primary care physicians in MASLD management, especially for patients without significant comorbidities.^41^ It underscores the need for strengthened advocacy and anti-stigma education in the primary care setting to ensure that such recommendations can be effectively implemented.

Overall, these results call for the integration of stigma-reduction strategies into MASLD care models, including interventions aimed at reducing stigma in healthcare settings,^41^ improving provider communication, reducing weight- and disease-related biases, empowering and supporting patients to reduce self-stigma, and managing the medical and psychosocial aspects of their disease and associated comorbidities. Such strategies may include stigma-aware communication training or social contact interventions^6^ for primary care providers, gender-sensitive counselling approaches, and routine assessment of stigma and self-stigma in MASLD clinics to identify individuals at increased risk of disengaging from care.

To our knowledge, this is the first study to comprehensively examine the relationship between self-stigma, perceived stigma, and care avoidance in MASLD while also exploring the determinants of self-stigma and perceived stigma separately in women and men. Specifically, this study provides the first international evidence disentangling perceived stigma and self-stigma and examining their gender-specific associations with care avoidance in MASLD. This study’s strength lies in its sample and detailed data collection. This study’s large sample size and the international nature of the sample enhance the generalisability of our findings. We were able to characterise two different dimensions of stigma and distinguish their associations with care avoidance while controlling for multiple potential confounders related to sociodemographic, geographic, and clinical status.

However, some limitations should be acknowledged. The study population consisted exclusively of individuals already engaged in care, primarily attending hepatology services. As recruited patients were those invited by their hepatologist/gastroenterologist and willing to participate, this may have introduced a selection bias. This could lead to an underestimation of stigma prevalence, as individuals avoiding hepatology/gastroenterology services are less likely to participate, thereby favouring patients with a better relationship with their healthcare providers. However, individuals more concerned by or sensitive to stigma may also have been more likely to participate in a survey focusing on stigma, which could partly counterbalance this effect. Therefore, the findings may not be fully generalisable to all individuals living with MASLD, particularly those not engaged in specialist care, suggesting that the stigma experience may be more widespread in this population. For example, among people with alcohol use disorders recruited within and outside healthcare settings, stigma was associated with a sixfold higher risk of care avoidance.^42^ Future research should aim to include individuals outside specialised care settings to obtain a more complete understanding of stigma and its effects on care avoidance across the full spectrum of patients with MASLD. The absence of specific measures of social support or living situation limits our ability to disentangle their independent contribution from stigma-related constructs in shaping care-seeking behaviours. Another limitation is that self-reported data on health outcomes and stigma may be subject to recall or social desirability bias,^43^ resulting in non-differential misclassification and underestimated associations (*i.e.* towards the null hypothesis) of the true impact of stigma on care avoidance.

To conclude, stigma is a major driver of care avoidance in MASLD, with distinct gender-specific patterns. Targeted strategies addressing self- and perceived stigma—through patient support, provider training, and public awareness—can improve early diagnosis and continuity of care. These findings inform health policy and highlight the need for future research on stigma among individuals not yet in specialist care, to develop equitable, gender-sensitive interventions and ensure timely access for all.

## Abbreviations

AOR, adjusted odds ratio; AUDIT-C, Alcohol Use Disorders Identification Test-Consumption; BMI, body mass index; CI, confidence interval; GNC, Global NASH/MASH Council; MASH, metabolic dysfunction-associated steatohepatitis; MASLD, metabolic dysfunction-associated steatotic liver disease; MENA, Middle East and North Africa; NA, not applicable; NAFLD, non-alcoholic fatty liver disease; NASH, non-alcoholic steatohepatitis; OR, odds ratio; SD, standard deviation; SSS-S, Self-Stigma Scale-Short; WCG, WIRB Copernicus Group.

## Authors’ contributions

Lead investigation, study design, project management, and critical writing and review of the manuscript: ZMY, PC, SZS.

Data management, statistical analysis, and critical writing and review of the manuscript: MS.

Critical writing and review of the manuscript: all the other authors.

## Data availability

Data may be available upon written request to the corresponding author.

## Financial support

This study was supported by the 10.13039/100008086Global NASH/MASH Council (GNC), Center for Outcomes Research in Liver Diseases, Washington, DC. No external funds were received to support this project.

## Conflicts of interest

ZMY has received research funding and/or serves as a consultant/advisor to Intercept, Cymabay, Boehringer Ingelheim, Ipsen, BMS, GSK, Novo Nordisk, Siemens, Madrigal, Merck, Aligos, Akero, Sanofi, and Abbott. ZMY has not received any funding for this work. NA has received grant/research support from 89bio, Akero Therapeutics, Arbutus Biopharma, AstraZeneca, BioAge, Boehringer Ingelheim, Bristol Myers Squibb, Corcept Therapeutics, Galectin Therapeutics, Genentech, Gilead Sciences, Hepagene Therapeutics, Intercept Pharmaceuticals, Inventiva Pharma, Ionis Pharmaceuticals, Ipsen, Lilly, Madrigal Pharmaceuticals, Merck, NGM Biopharmaceuticals, NorthSea Therapeutics, Novo Nordisk, Perspectum, Pfizer, PharmaIN, Poxel, Regeneron, Viking Therapeutics, and Zydus Pharmaceuticals; reports speaker fees from AbbVie, AstraZeneca, Echosens, Gilead Sciences, Ipsen, Madrigal Pharmaceuticals, Novo Nordisk, and Perspectum; and reports consulting fees from 89bio, AbbVie, Akero, Boehringer Ingelheim, Echosens, Fibronostics, Gilead Sciences, HistoIndex, Intercept Pharmaceuticals, Ipsen, LiverRight, Madrigal Pharmaceuticals, NorthSea Therapeutics, Novo Nordisk, Perspectum, Pfizer, Regeneron, and Sonic Incytes. SKR is an advisory board member of Novo Nordisk. AKS serves as a consultant on the SBIR grant for Pleiogenic Pharma; is on the advisory boards of Orphalan, Disc Medicine, Ipsen, and Madrigal; and speaks and writes for Medscape, CLD Foundation, Expert Perspectives, Gastro Endo News, DynaMed, Medical Education Speakers Network, UptoDate, Madrigal Pharma, and Practice Point. AKS received funding grants from NIAAA-NIH, NIGMS-NIH, and DHHS. TK received lecture fees from ASKA Pharmaceutical Co., Ltd., Taisho Pharmaceutical Co., Ltd., Kowa Company, Ltd., AbbVie GK, Eisai Co., Ltd., EA Pharma Co., Ltd., Nippon Boehringer Ingelheim Co., Ltd., Sumitomo Pharma Co., Ltd., Novo Nordisk Pharma Ltd., Otsuka Pharmaceutical Co., Ltd., and Janssen Pharmaceutical K.K. YY serves as a consultant for Zydus and an adviser for Novo Nordisk. MA has consulted for AstraZeneca and Inventiva and served as a speaker for Novo Nordisk and Siemens. He receives support from the Agencia Nacional de Investigación y Desarrollo de Chile (Fondecyt 1241450). SF holds a senior clinical investigator fellowship from the Research Foundation Flanders (FWO) (1802154N). His institution has received grants from Astellas, Falk Pharma, Genfit, Gilead Sciences, GlympsBio, Janssen Pharmaceutica, Inventiva, Merck Sharp & Dohme, Pfizer, and Roche. He has acted as a consultant for AbbVie, Actelion, Aelin Therapeutics, AgomAb, Aligos Therapeutics, Allergan, Alnylam, Astellas, AstraZeneca, Atheneum Partners GmbH, Bayer, Boehringer Ingelheim, Bristol Meyers Squibb, Byondis, CSL Behring, Coherus, Echosens, Decisive Consulting, Dr. Falk Pharma, Eisai, Enyo, Galapagos, Galmed, Genentech, Genfit, Genflow Biosciences, Gilead Sciences, Intercept, Inventiva, Janssen Pharmaceutica, PRO.MED.CS Praha, Julius Clinical, Madrigal, MedImmune, Merck Sharp & Dohme, Mursla Bio, NGM Bio, Novartis, Novo Nordisk, Promethera, Roche, Siemens Healthineers, Silengenics, V4Cure, and Weatherden. SF has been lecturer for AbbVie, Allergan, Bayer, Eisai, Genfit, Gilead Sciences, Janssen Cilag, Intercept, Inventiva, Merck Sharp & Dohme, Novo Nordisk, Promethera, and Siemens. PB serves as a consultant for Biotest, Kedrion, Sandoz, Chiesi Farmaceutici, GSK, Novo Nordisk, and Ipsen. LY received research support from AbbVie, Abbott Diagnostics, Boehringer Ingelheim, BMS, Gilead, Novo Nordisk, and Roche Diagnostics; served as a consultant for AbbVie, Abbott Diagnostics, BMS, Gilead, Novo Nordisk, Roche, and Roche Diagnostics; and served as a speaker for AbbVie, BMS, Eisai, Gilead, Roche, and Roche Diagnostics. LY has not received any funding for this work. VW has served as a consultant or advisory board member for AbbVie, Altimmune, AstraZeneca, Boehringer Ingelheim, Echosens, Eli Lilly, Gilead Sciences, Merck, Novartis, Novo Nordisk, Pfizer, Roche Diagnostics, TARGET PharmaSolutions; a speaker for Abbott, AbbVie, AstraZeneca, Echosens, Gilead Sciences, and Novo Nordisk; an independent non-executive director for Furui; and a co-founder for Illuminatio Medical Technology. He has received a research grant from Gilead Sciences. CWS has received honoraria as a speaker for Novo Nordisk and Sanofi. WKC has served as a consultant or advisory board member for Abbott, AbbVie, Boehringer Ingelheim, IPSEN, Kowa, Novo Nordisk, Roche, and Zuellig Pharma and as a speaker for Abbott, Echosens, Hisky Medical, Novo Nordisk, Roche, and Viatris. WKC received research grants from Abbott and Roche. BL reports serving as a speaker for Madrigal and Novo Nordisk and as a consultant for Madrigal, Novo Nordisk, and 89bio. MEK reports having served as an investigator, speaker, and/or advisory board member for AstraZeneca, Roche, MSD, AbbVie, Eva, Multicare, Mash Premier, Takeda, Organon, AUG, Inspire, HSO, Gilead, Janssen, Intercept, Rameda, FPI, Ipsen, Onxeo, Sanofi, Sandoz, Al Andalus Pharma, Minapharm, Pharco, Zeta, Alfa Cure, Bayer, Oncoustics, PDC, Acino, Opella, Spimaco, and Novo Nordisk. LC reports consulting fees from Boehringer Ingelheim, Boston Pharmaceutical, Echosens, Gilead, GSK, Madrigal, MSD, Novo Nordisk, Pfizer, Sagimet, and Siemens Healthineers and speaker fees from AstraZeneca, Boehringer Ingelheim, Echosens, Gilead, Madrigal, and Novo Nordisk. MLY received research support from Abbvie, Abbott Diagnostic, Boehringer Ingelheim, BMS, Gilead, Novo Nordisc and Roche diagnostics, served as a consultant of Abbvie, Abbott Diagnostic, BMS, Gilead, GSK, Novo Nordisc, Roche and Roche diagnostics, and a speaker of Abbvie, BMS, Eisai, Gilead, Novo Nordisc, Roche and Roche diagnostics.The remaining authors declare no conflicts of interest that pertain to this work.

Please refer to the accompanying ICMJE disclosure forms for further details.

## References

[1] Cossins B.C., van den Munckhof I., Rutten J.H.W. (2023). Sex-specific association between adipose tissue inflammation and vascular and metabolic complications of obesity. J Clin Endocrinol Metab.

[2] Yan T., Zhang X., Wong T. (2024). Sex differences in adverse liver and nonliver outcomes in steatotic liver disease. JAMA Netw Open.

[3] Balakrishnan M., Patel P., Dunn-Valadez S. (2021). Women have a lower risk of nonalcoholic fatty liver disease but a higher risk of progression vs men: a systematic review and meta-analysis. Clin Gastroenterol Hepatol.

[4] DiStefano J.K. (2020). NAFLD and NASH in postmenopausal women: implications for diagnosis and treatment. Endocrinology.

[5] Younossi Z.M., AlQahtani S.A., Funuyet-Salas J. (2024). The impact of stigma on quality of life and liver disease burden among patients with nonalcoholic fatty liver disease. JHEP Rep.

[6] Corrigan P.W., Morris S.B., Michaels P.J. (2012). Challenging the public stigma of mental illness: a meta-analysis of outcome studies. Psychiatr Serv.

[7] Link B.G., Phelan J.C. (2006). Stigma and its public health implications. Lancet.

[8] Younossi Z.M., Alqahtani S.A., Alswat K. (2024). Global survey of stigma among physicians and patients with nonalcoholic fatty liver disease. J Hepatol.

[9] Carol M., Perez-Guasch M., Sola E. (2022). Stigmatization is common in patients with non-alcoholic fatty liver disease and correlates with quality of life. PLoS One.

[10] Hughes A.M., Flint S.W., Clare K. (2024). Demographic, socioeconomic and life-course risk factors for internalized weight stigma in adulthood: evidence from an English birth cohort study. Lancet Reg Health Eur.

[11] Sattler K.M., Deane F.P., Tapsell L. (2018). Gender differences in the relationship of weight-based stigmatisation with motivation to exercise and physical activity in overweight individuals. Health Psychol Open.

[12] Just-Norregaard V., Andreasen P., Bruun J.M. (2025). Weight stigma in adolescents: sex differences, prevalence, emotional impact, and associations with BMI increases—a retrospective cohort study. BMC Public Health.

[13] Brewis A.A. (2014). Stigma and the perpetuation of obesity. Soc Sci Med.

[14] Himmelstein M.S., Puhl R.M., Quinn D.M. (2019). Overlooked and understudied: health consequences of weight stigma in men. Obesity.

[15] Lonardo A., Nascimbeni F., Ballestri S. (2019). Sex differences in nonalcoholic fatty liver disease: state of the art and identification of research gaps. Hepatology.

[16] Kim H., Kim S.E., Sung M.K. (2025). Sex and gender differences in obesity: biological, sociocultural, and clinical perspectives. World J Mens Health.

[17] Tomiyama A.J., Carr D., Granberg E.M. (2018). How and why weight stigma drives the obesity ‘epidemic’ and harms health. BMC Med.

[18] Tudor K., Tearne S., Jebb S.A. (2021). Gender differences in response to an opportunistic brief intervention for obesity in primary care: data from the BWeL trial. Clin Obes.

[19] Prunty A., Hahn A., O’Shea A. (2023). Associations among enacted weight stigma, weight self-stigma, and multiple physical health outcomes, healthcare utilization, and selected health behaviors. Int J Obes (Lond).

[20] Cuauro S.E., Santos N., Andrade E. (2023). Internalized weight stigma and weight discrimination: associations with quality of life and psychosocial impairment in a sample living with food insecurity. Int J Environ Res Public Health.

[21] Golay P., Martinez D., Silva B. (2022).

[22] Vaughn-Sandler V., Sherman C., Aronsohn A. (2014). Consequences of perceived stigma among patients with cirrhosis. Dig Dis Sci.

[23] Alguwaihes A.M. (2025). Gender differences in type 1 diabetes management and mental health burden: findings from a national survey in Saudi Arabia. J Clin Med.

[24] Sims T.J., Kapoor R., Chinthammit C. (2025). Relationship between weight bias internalization, diabetes stigma, and perceptions of healthcare interactions among people with type 2 diabetes. Diabetes Ther.

[25] Chatmon B.N. (2020). Males and mental health stigma. Am J Mens Health.

[26] Schlichthorst M., Sanci L.A., Pirkis J. (2016). Why do men go to the doctor? Socio-demographic and lifestyle factors associated with healthcare utilisation among a cohort of Australian men. BMC Public Health.

[27] Guglielmi V., Dalle Grave R., Leonetti F. (2024). Female obesity: clinical and psychological assessment toward the best treatment. Front Endocrinol (Lausanne).

[28] Phelan S.M., Bauer K.W., Bradley D. (2022). A model of weight-based stigma in health care and utilization outcomes: evidence from the learning health systems network. Obes Sci Pract.

[29] Inglis G., Sosu E., McHardy F. (2025). Testing the associations between poverty stigma and mental health: the role of received stigma and perceived structural stigma. Int J Soc Psychiatry.

[30] Earnshaw V.A., Quinn D.M. (2012). The impact of stigma in healthcare on people living with chronic illnesses. J Health Psychol.

[31] Carrieri P., Mourad A., Marcellin F. (2022). Knowledge of liver fibrosis stage among adults with NAFLD/NASH improves adherence to lifestyle changes. Liver Int.

[32] Hiscock R., Bauld L., Amos A. (2012). Socioeconomic status and smoking: a review. Ann N Y Acad Sci.

[33] Lawrence D., Hafekost J., Hull P. (2013). Smoking, mental illness and socioeconomic disadvantage: analysis of the Australian national survey of mental health and wellbeing. BMC Public Health.

[34] Noble N., Paul C., Turon H. (2015). Which modifiable health risk behaviours are related? A systematic review of the clustering of Smoking, Nutrition, Alcohol and Physical activity (‘SNAP’) health risk factors. Prev Med.

[35] David J.C., Fonte D., Sutter-Dallay A.L. (2024). The stigma of smoking among women: a systematic review. Soc Sci Med.

[36] Madawala S., Enticott J., Sturgiss E. (2023). The impact of smoking status on anticipated stigma and experience of care among smokers and ex-smokers with chronic illness in general practice. Chronic Illn.

[37] Rai S.S., Syurina E.V., Peters R.M.H. (2020). Non-communicable diseases-related stigma: a mixed-methods systematic review. Int J Environ Res Public Health.

[38] Figueroa D.G., Murley W.D., Parker J.E. (2025). Weight stigma and mental health symptoms: mediation by perceived stress. Front Psychiatry.

[39] Lee K.M., Hunger J.M., Tomiyama A.J. (2021). Weight stigma and health behaviors: evidence from the Eating in America Study. Int J Obes (Lond).

[40] Egea Sancho M., Ramirez-Manent J.I., Lopez P.J.T. (2026). Associations of sociodemographic factors, healthy habits, and weight stigma with non-alcoholic fatty liver disease risk in obese workers: findings from a large occupational cohort. Gastroenterol Hepatol.

[41] Karlsen T.H., Sheron N., Zelber-Sagi S. (2022). The EASL-Lancet Liver Commission: protecting the next generation of Europeans against liver disease complications and premature mortality. Lancet.

[42] Carrieri P., Barre T., Bureau M. (2022). Stigma and care avoidance in people with unhealthy alcohol use: a call for research and action. J Hepatol.

[43] Latkin C.A., Edwards C., Davey-Rothwell M.A. (2017). The relationship between social desirability bias and self-reports of health, substance use, and social network factors among urban substance users in Baltimore, Maryland. Addict Behav.

