# Supplementary material for: Gender differences in stigma and care avoidance among people living with MASLD: An international survey
Source: JHEP Rep. 2026 Sep 2;8(11):101987. doi: 10.1016/j.jhepr.2026.101987 (PMC13694871; doi:10.1016/j.jhepr.2026.101987)
Supplement: Supplementary file 2 [file mmc1.docx]

**Journal of Hepatology Reports**

**CTAT methods**

Tables for a “Complete, Transparent, Accurate and Timely account” (CTAT) are now mandatory for all revised submissions. The aim is to enhance the reproducibility of methods.

- Only include the parts relevant to your study
- Refer to the CTAT in the main text as ‘Supplementary CTAT Table’
- Do not add subheadings
- Add as many rows as needed to include all information
- Only include one item per row

**If the CTAT form is not relevant to your study, please outline the reasons why:**

Secondary analysis of survey data.

1.1 **Antibodies**

| **Name** | **Citation** | **Supplier** | **Cat no.** | **Clone no.** |
| --- | --- | --- | --- | --- |
|  |  |  |  |  |

1.2 **Cell lines**

| **Name** | **Citation** | **Supplier** | **Cat no.** | **Passage no.** | **Authentication test method** |
| --- | --- | --- | --- | --- | --- |
|  |  |  |  |  |  |

1.3 **Organisms**

| **Name** | **Citation** | **Supplier** | **Strain** | **Sex** | **Age** | **Overall n number** |
| --- | --- | --- | --- | --- | --- | --- |
|  |  |  |  |  |  |  |

1.4 **Sequence based reagents**

| **Name** | **Sequence** | **Supplier** |
| --- | --- | --- |
|  |  |  |

1.5 **Biological samples**

| **Description** | **Source** | **Identifier** |
| --- | --- | --- |
|  |  |  |

1.6 **Deposited data**

| **Name of repository** | **Identifier** | **Link** |
| --- | --- | --- |
|  |  |  |

1.7 **Software**

| **Software name** | **Manufacturer** | **Version** |
| --- | --- | --- |
| **SAS 9.4** | **SAS Institute** | **9.4** |

1.8 **Other (e.g. drugs, proteins, vectors etc.)**

1.9 **Please provide the details of the corresponding methods author for the manuscript:**

Zobair M. Younossi, MD, MPH

The Global NASH/MASH Council, Center for Outcomes Research in Liver Diseases

2411 I Street, Washington DC 20037

Phone: +1 (703) 283-1638; Email: Zobair.Younossi@cldq.org

**2.0	Please confirm for randomised controlled trials all versions of the clinical protocol are included in the submission. These will be published online as supplementary information.**

**n/a**
